# Exosomes: Potential Biomarkers and Functions in Head and Neck Squamous Cell Carcinoma

**DOI:** 10.3389/fmolb.2022.881794

**Published:** 2022-06-14

**Authors:** Ting Li, Juan Li, Haitao Wang, Jiayu Zhao, Mingze Yan, Hongjiang He, Shan Yu

**Affiliations:** ^1^ Department of Head and Neck Surgery, Harbin Medical University Cancer Hospital, Harbin, China; ^2^ Department of Pathology, Second Affiliated Hospital of Harbin Medical University, Harbin, China; ^3^ Thoracic Surgery Branch, National Cancer Institute, National Institutes of Health, Bethesda, MD, United States

**Keywords:** head and neck squamous cell carcinoma, exosome, biomarkers, therapeutic target, drug carrier

## Abstract

Head and neck squamous cell carcinoma (HNSCC), originating from the mucosal epithelial cells of the oral cavity, pharynx, and larynx, is a lethal malignancy of the head and neck. Patients with advanced and recurrent HNSCC have poor outcomes due to limited therapeutic options. Exosomes have active roles in the pathophysiology of tumors and are suggested as a potential therapeutic target of HNSCC. Exosomes in HNSCC have been intensively studied for disease activity, tumor staging, immunosuppression, and therapeutic monitoring. In this review, the biological mechanisms and the recent clinical application of exosomes are highlighted to reveal the potential of exosomes as biomarkers and therapeutic targets for HNSCC.

## Introduction

Head and neck squamous cell carcinoma (HNSCC), as common malignant tumors of the head and neck, originates from the mucosal epithelial cells of the oral cavity, pharynx, and larynx (Johnson et al., 2020). Epidemiological studies have shown that 75–85% of HNSCCs are caused by smoking and alcohol consumption (Machiels et al., 2020). Currently, various therapeutic interventions, including surgery, radiotherapy, chemotherapy, and immunotherapy, are applied to improve the HNSCC patient outcomes (Johnson et al., 2020). Despite the advances in the comprehensive treatments of HNSCC, the 5-years overall survival rate of HNSCC patients remains lower than 60% (Bray et al., 2018). About 50% of patients with locally advanced HNSCC develop disease recurrence and drug resistance after initial treatment, resulting in a poor prognosis with a median survival of about 12 months (Leeman et al., 2017; Ionna et al., 2021; Kozłowska et al., 2021). Therefore, there is an urgent need to develop effective therapeutic targets for HNSCC (Martin et al., 2014). Investigations have shown that exosomes are significantly associated with HNSCC in tumorigenesis, development and other cancer hallmarks (Ebnoether and Muller, 2020). In addition, HNSCC exosomes are involved in immune regulation, and drug resistance (Cheng et al., 2019; Xiao et al., 2019). Recently, tremendous efforts also reveal exosomes may be a new therapeutic target for HNSCC cure (Syn et al., 2017).

## Knowledge in Composition and Secretion of Exosomes

As “natural nanoparticles” produced by plants, microbes, or the body’s cells, exosomes are a type of extracellular vesicles (EVs) that differ from other larger types of EVs in their size as well as their biogenesis pathways (Srivastava et al., 2022). Exosomes, the largest subtype of EVs from endosomes, are typically in the 40–160 nm (average-100 nm) diameter range (Kalluri and LeBleu, 2020). Exosome membrane has a phospholipid bilayer like the cytoplasmic membrane (Dai et al., 2020). (Table 1 summarizes the comparison of exosome membrane and cytoplasmic membrane.) Exosomes are derived from the endocytosis compartment of parental cells and widely exist in all body fluids such as blood, saliva, urine, and cerebrospinal fluid. Exosomes could be released by all cell types and recon sided as a newly found pathway for cell-to-cell communication (Simpson et al., 2008; Milane et al., 2015; Wiklander et al., 2019). Recent data support that exosomes carry and deliver biologically active components and regulate a wide range of physiological and pathological events, including cancers (Peinado et al., 2011; Dai et al., 2020; Kalluri and LeBleu, 2020).

**TABLE 1:** Comparison of exosome membrane and cytoplasmic membrane.

	Composition	Biological functions	Refs
Exosome Membrane	a. Lipid rafts (Sphingolipids, cholesterol, phosphatidylserine, ceramides) b. Tetraspanins (CD9, CD63, CD81) c. Transmembrane proteins (FasL, PD-L1, CTLA-4) d. Membrane trafficking proteins (Annexins, Rabs) e. Immuno-regulatory molecules (MHCⅠ, MHCⅡ) f. Integrins	a. Angiogenesis b. Apoptosis c. Antigen presentation d. Inflammation e. Biomarkers f. Receptor-mediated endocytosis g. Cell proliferation and differentiation	Gurunathan et al. (2019)
Cytoplasmic Membrane	a. Glycerol phospholipids, sphingomyelins, and sterols b. Extrinsic membrane protein (peripheral membrane protein) c. Intrinsic membrane protein (integral membrane protein) d. lipid anchored protein	a. Stable internal environment b. Selective transportation of materials c. Recognition sites for transmembrane transmission of intracellular and intracellular information d. The binding site of enzyme e. Mediating cell-to-cell and cell-to-extracellular matrix links f. Formation of different cell surface transformation structures g. Therapeutic targets	Engelman (2005); Grecco et al. (2011)

**FIGURE 1:**
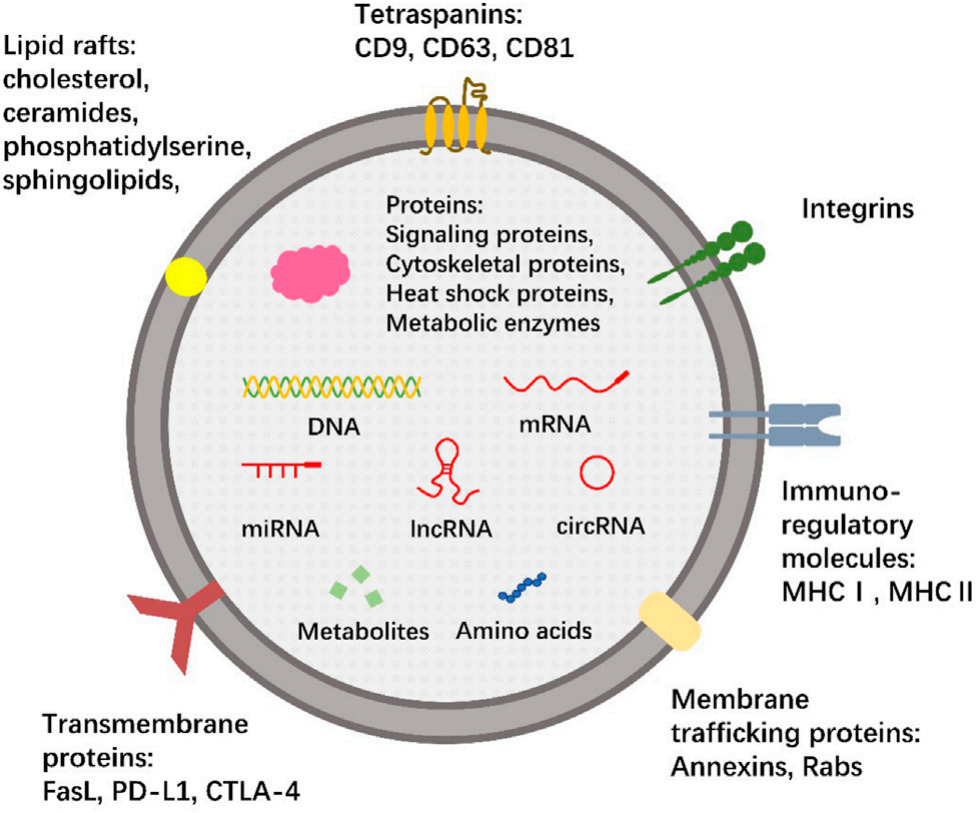
The composition of exosomes. The membrane of exosomes, like cells, is composed of the phospholipid bilayer. The membrane surface contains a wide range of tetraspanins (CD9, CD63, CD81), transmembrane proteins (FasL, PD-L1, CTLA-4), membrane trafficking proteins (Annexins, Rabs), integrins and immuno-regulatory molecules (MHCⅠ, MHCⅡ) that bind specific peptide chains. There are also several lipid rafts involved, such as phosphatidylserine, sphingolipids, cholesterol, and ceramides. Exosomes contain a variety of nucleic acids, including not only DNA and mRNA, but also many ncRNAs, including miRNA, lncRNA and circRNA. Some proteins, amino acids, and metabolites can also be encapsulated in exosomes. This is a typical but not comprehensive representation of exosomes, and no single exosome is expected to contain all or even most of the bioactive molecules shown.

The bioactive components of exosomes include nucleic acids, proteins, lipids, amino acids, and metabolites are known to be important for their functions (Kalluri and LeBleu, 2020). Characteristic marker proteins are present on or within exosomes, and their composition is highly dependent on their source and cell state (rest, stimulation, inhibition, or transformation) (Bang and Thum, 2012; Jan et al., 2021). In addition, non-coding RNAs (ncRNAs) are found in exosomes, including microRNA (miRNA), long non-coding RNA (lncRNA), and circular RNA (circRNA). Nucleic acid content in exosomes is proposed to be involved in the occurrence and development of cancer by inducing carcinogenic transformation and transfer of specific cancer genetic material (Kalluri and LeBleu, 2016). Exosomes have lipid rafts that are different from the parental plasma membrane and are rich in unique lipids (Trajkovic et al., 2008; Record et al., 2014). Evidence has shown that exosomes with high prostaglandin PGE2 content were involved in promoting tumor growth and immune escape (Xiang et al., 2009). We summarize the composition of exosomes in Figure 1.

**FIGURE 2:**
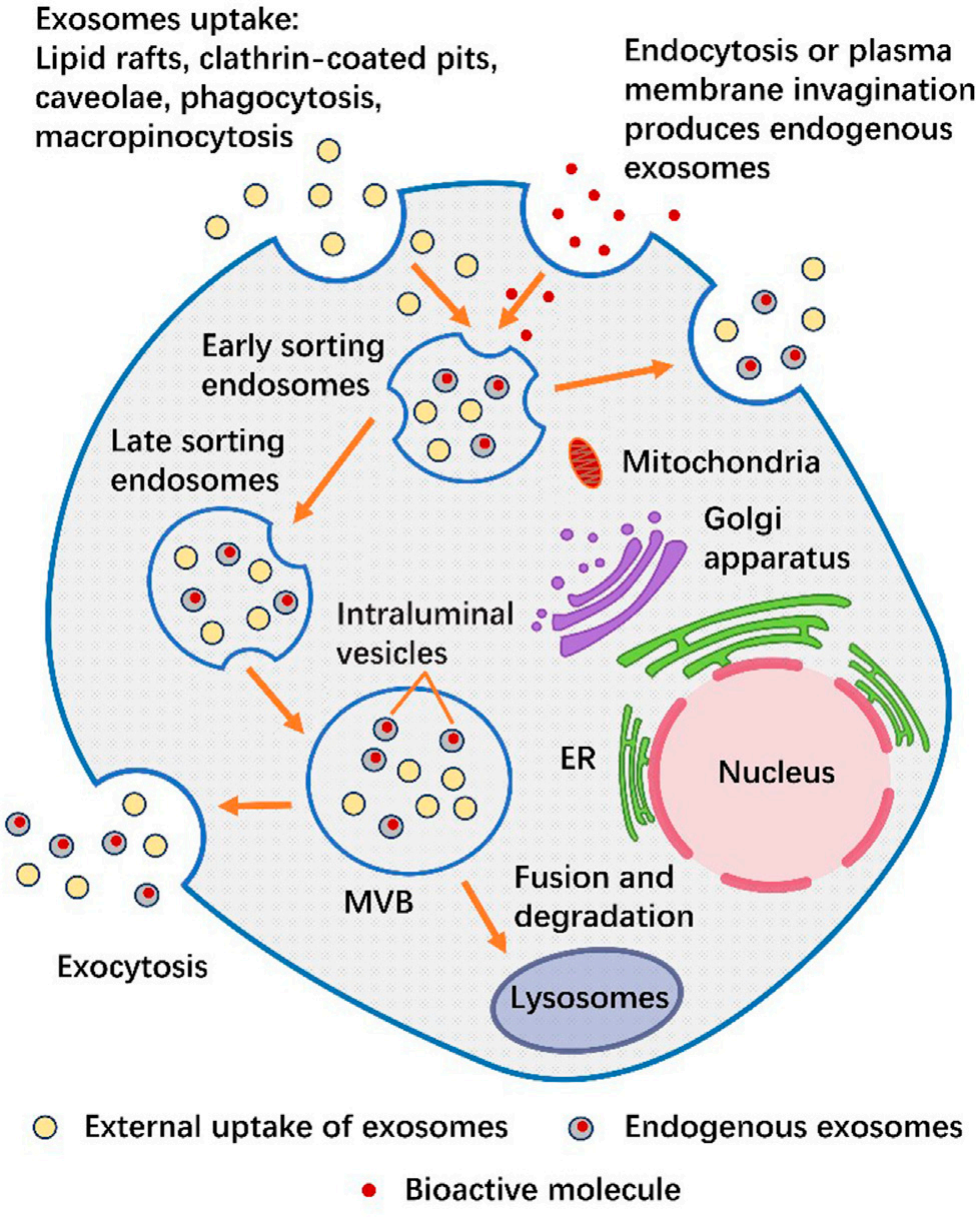
The secretion process of exosomes. Exosomes can either produce endogenous exosomes through cytocytosis or plasma membrane invagination to take up bioactive molecules (nucleic acids, proteins, lipids, amino acids, or metabolites) (gray), or they can be secreted from the outside through lipid rafts, clathrin-coated pits, caveolae, phagocytosis or macropinocytosis in the form of external uptake of exosomes (yellow). The mixture of the two is aggregated in ESEs. During this period, the exosomes inside can either fuse with the plasma membrane and release exosomes outside the cell, or fuse downward to form LSEs and then form MVB. One part of MVB is released to the extracellular environment through exocytosis, while the other part is Fusion and degradation in the lysosome.

The production and release of exosomes are known as an orderly process (Vlassov et al., 2012). Production of exosomes begins with the uptake of extracellular components (functional proteins, RNA, lipids, etc.) through plasma membrane invagination or endocytosis. Exosomes can also be ingested by lipid rafts, clathrin-coated pits, caveolae, phagocytosis, and micropinocytosis (Commisso et al., 2013; Kamerkar et al., 2017; Kalluri and LeBleu, 2020). The mixture of the two polymerizes in early sorting endosomes (ESEs), most of which form late sorting endosomes (LSEs), and a few could directly form exosomes and be released into the cytoplasm (Wen et al., 2019). LSEs change their contents through secondary invagination and accumulate in lumen to form a multivesicular body (MVB) containing many intraluminal vesicles (ILVs) (Zhao et al., 2020a; Kalluri and LeBleu, 2020). The endosomal-sorting complex required for transport (ESCRT) is the main mechanism for the formation of ILVs and the sorting of specific goods (Oggero et al., 2019). Starting from ESCRT-0, cargo specific ILVs are formed through initiation, recruitment, assembly, action and disassembly, which eventually become MVB (Henne et al., 2011; Colombo et al., 2013). MVBs can be fused with lysosomes for degradation. On the other hand, ILVs can release exosomes from MVBs into the extracellular environment through exocytosis (Fais et al., 2013; Zhao et al., 2020a; Kalluri and LeBleu, 2020; Jan et al., 2021). We summarize the secretion process of exosomes in Figure 2.

## Function of Exosomes in HNSCC

Exosomes perform important extracellular functions through communication between tumors and surrounding stromal tissues, including interactions with the cellular microenvironment through immune-mediated, morphogenesis signaling and cell recruitment (Peinado et al., 2011; Donnarumma et al., 2017). In oncology, tumor cells secrete a large number of exosomes. The molecular cargo of tumor-derived exosomes (TEXs) is enriched with some key molecular characteristics and can serve at least to some extent as a substitute for parent tumor cells (Whiteside, 2016a). They are involved in the occurrence, progression, metastasis, and in immune escape (Zhao et al., 2020a). In a recent study, Pang et al. found that exosomal CMTM6 secreted by oral squamous cell carcinoma (OSCC) cells induced polarization of M2-like macrophages through the ERK1/2 signaling pathway and strongly promoted proliferation, invasion, and migration of OSCC cells (Bellmunt et al., 2019; Pang et al., 2021). Mutschelknaus et al. found that a low radiation dose of 3 Gy did not lead to enhanced migration of HNSCC cells, while exosomes isolated from HNSCC cells enhanced chemotaxis under 6 and 9 Gy radiation, indicating that the pro-migration response of exosomes was dose-dependent. Further investigation of the mechanism revealed that exosomes isolated from HNSCC cells promoted phosphorylation of mTOR, a downstream target of AKT signaling, phosphorylation of rpS6, and induce the AKT downstream targets release (such as MMP2 and MMP9) (Mutschelknaus et al., 2017). Epstein-Barr Virus (EBV) is one of the risk factors for OSCC. It had been found that EBER-1 (small RNA-1 encoded by EBV) was released into exosomes from EBV-infected OSCC cells and internalized by adjacent stromal macrophages. Exosomes carrying EBER-1 could induce up-regulated expression of indoleamine 2,3 dioxygenase (IDO) through RIG-I signaling mediated inflammatory pathways. Activation of IDO in response to EBER expression of monocyte-derived macrophages could be a key step in inhibiting T cell response by upregulation of kynurenine. Impaired T cell response prevents the transformation of transformed oral cells, which promotes the occurrence and development of OSCC (Burassakarn et al., 2021).

## Exosomes as Diagnostic and Prognostic Biomarkers for HNSCC

Traditional cancer diagnosis methods, such as endoscopy, computed tomography, X-ray, positron emission computed tomography, magnetic resonance imaging, and invasive biopsy, are neither suitable for use in large populations nor repeated screening (Park et al., 2017). Biomarkers are becoming a promising strategy for cancer diagnosis and treatment effect evaluation. However, the high heterogeneity of HNSCC is a challenge in its identification. Since HNSCC is an enthusiastic producer of exosomes, the level of exosomes in HNSCC patients was used as diagnostic and prognostic biomarkers. Exosomes are highly stable and easily collected from body fluids including urine or blood through non-invasive or minimally invasive methods. Compared with proteins or small molecules, exosomes are located at the level of organelles with functionally heterogeneous molecules representing the complexity of parent cells (Maia et al., 2018; Pullan et al., 2019; Nam et al., 2020). Previous studies have found higher levels of exosomes in the plasma of HNSCC patients compared to healthy donors. Furthermore, it was found that the protein levels in exosomes isolated from the plasma of HNSCC patients were sufficient to distinguish stage I/II patients from stage III/IV patients (Ludwig et al., 2017). In terms of prognosis, total exosome levels, TEX/total exosome ratio, and phenotypic characteristics of exosomes from TEX or T cells showed the ability to distinguish HNSCC patients who responded to or did not respond to tumor therapy (Theodoraki et al., 2019). This evidence suggests that HNSCC exosomes can be used as “molecular markers” to provide relevant information about the diagnosis and prognosis of HNSCC.

## Protein

The exosomes secreted by HNSCC are rich in many invasive molecules, and the specific proteins can be detected and used as biomarkers to monitor the disease progression of HNSCC (Zhao et al., 2020a). HSP90 levels in exosomes were elevated in OSCC cells with lymph node metastasis and that high levels of HSP90 were associated with poor prognosis, especially OSCC patients with metastasis (Ono et al., 2018). One study, based on the seventh edition of UICC (Union for International Cancer Control) tumor-node-metastasis (TNM) classification of malignant tumors, demonstrated that serum exosome lysyl oxidase-like 2 (LOXL2) levels were significantly higher in stage I/II HNSCC patients than in healthy volunteers and stage III HNSCC patients. LOXL2 promoted tumor progression by remodeling HNSCC extracellular matrix and increasing epithelial-mesenchymal transition (EMT). In addition, previous studies had verified that LOXL2 mRNA expression was up-regulated in metastatic HNSCC cells compared with non-metastatic cells (Demory Beckler et al., 2013). Taken together, these results suggest that serum exosome LOXL2 levels are only associated with early-stage HNSCC, and may serve as biomarkers for early diagnosis and a potential target for therapeutic intervention (Cox et al., 2016; Sanada et al., 2020). Both protein and mRNA levels of Annexin A1 (ANXA1) were down-regulated in HNSCC. Knockdown ANXA1 reduced the production of exosomes in HNSCC cell lines and the number of associated exosomes phosphorylated with epidermal growth factor receptor (EGFR) (Raulf et al., 2018). The increased EGFR phosphorylation is associated with poor prognosis (Patel et al., 2005; Hiraishi et al., 2006). Serum Alix level in patients with OSCC lymph node metastasis was significantly higher than that in healthy controls, which was related to the stage of OSCC. The elevation of Alix level outside saliva was not associated with OSCC stage. Serum exosome Alix level has high specificity and positive predictive value, which can be used as a prognostic indicator of treatment response (Nakamichi et al., 2021).

## miRNA

As tumor suppressors or oncogenes, miRNAs have been shown to regulate cell differentiation, proliferation, and apoptosis, and have a role in promoting tumor development (Nassar et al., 2017). Due to the lack of endogenous RNase, many investigators have found a high concentration of multiple functional oncogenic miRNAs in HNSCC exosomes (Yang et al., 2020a). Coon, et al. demonstrated that in OSCC cells, miR-365 was much higher than the level required for basic maintenance of cell function. Overexpressed miR-365 is delivered to the OSCC exosomes and has the potential to serve as a potential biomarker for OSCC for saliva diagnosis and other types of liquid biopsies (Coon et al., 2020). Regarding human papillomavirus (HPV) infected HNSCC, exosomes rich in miR-9 could transform polarized macrophages into M1 type by down-regulating PPAR δ. The increased radiosensitivity of HPV(+) HNSCC demonstrated that miR-9 expression in exosomes could be a potential biomarker and therapeutic approach (Tong et al., 2020). Another study found that miR-941 was detected in serum exosomes of laryngeal squamous cell carcinoma (LSCC), quantitative reverse transcription PCR and ROC curve analysis showed that up-regulated level of miR-941 promoted cell proliferation and invasion (Zhao et al., 2020b).

Tumor-associated fibroblasts (CAFs) are the most abundant cells in the microenvironment of tumor cells. They release exosomes that alter the tumor microenvironment with a variety of proteins and miRNAs (Donnarumma et al., 2017). The disorder of miRNA is a characteristic manifestation of the transformation of normal fibroblasts into CAF according to cancer status (Yang et al., 2017). It has been reported that, as a mediator involved in CAF-OSCC cell communication, miR-382-5p could transport miR-382-5p from CAF-derived exosomes to OSCC cells, thereby promoting the invasion and metastasis of OSCC (Sun et al., 2019). Another study found that miR-14 expression was significantly down-regulated in CAF-derived exosomes isolated from OSCC tissues, further leading to activation of the Wnt/β-catenin signaling pathway and EMT, thereby enhancing OSCC invasion and metastasis (Jin et al., 2020).

Hypoxia is common in tumor tissues and is characteristic of the tumor microenvironment, which enhances the release of TEX. As tumor tissues develop in an anoxic environment, they not only further exacerbate anoxic conditions but are also prone to drug resistance under such conditions (Bhandari et al., 2019). In addition, hypoxia could stimulate the production of miR-21-rich exosomes in OSCC cells, which was directly regulated by HIF-1α and HIF-2α. Those exosomes rich in miR-21 were delivered to non-hypoxic cells to promote pre-metastatic phenotypes and played an important role in the migration and invasion of OSCC. Notably, circulating exosome miR-21 was found to have the potentials for the diagnosis and prognosis of OSCC (Li et al., 2016).

miRNAs have been extensively studied in saliva to regulate various pathogenic processes of cancer through their interactions with target mRNAs (Topkas et al., 2012; Schulz et al., 2013). Exosome protein in saliva is less than that in blood, which is easy to collect and non-invasive, greatly simplifying the identification procedure, and is an excellent method for monitoring OSCC (Topkas et al., 2012; Schulz et al., 2013). miRNAs in saliva are potential biomarkers for a variety of diseases, including OSCC (Brinkmann et al., 2011). For example, He et al. confirmed that the expression of miR-24-3p in salivary exosomes of OSCC patients was significantly higher than that of normal individuals, which could be distinguished from normal individuals with high accuracy. Further data analysis showed that miR-24-3p could inhibit PER1 by directly targeting its 3′-UTR, thereby promoting the proliferation of OSCC cells (Gai et al., 2018).

Drug resistance is a major challenge with molecular mechanisms unknown for HNSCC treatment (Zhao et al., 2020a). Exogenous drug resistance caused by crosstalk between the tumor and tumor microenvironment (TME) can be mediated by miRNA exosome transfer (Valadi et al., 2007; Nwabo et al., 2017). Qin et al. found that CAFs were inherently resistant to cisplatin and are transmitted from CAF to tumor cells through exosome miR-196a, which significantly improved cell proliferation and survival rate of HNSCC cells. Then, exosome miR-196a endowed HNSCC with a cytochemical resistance phenotype by binding downstream target genes CDKN1B and ING5 in the HNSCC microenvironment. Exosomes of CAF or depletion of exosomes miR-196a restored the sensitivity of HNSCC to cisplatin. In addition, Kaplan-Meier analysis and Cox regression analysis showed that plasma high expression of exosome miR-196a in HNSCC patients was associated with poor overall survival and was a valuable prognostic factor. These results indicate that exosome miR-196a can be used as a predictor of cisplatin resistance and an important prognostic factor in HNSCC patients (Qin et al., 2019). Other experimental results showed that cisplatin-resistant OSCC cells secreted higher levels of exosome miR-21 than those secreted from OSCC patients. Those exosomes could induce cisplatin resistance in OSCC cells by increasing the expression of miR-21 and thereby reducing the levels of downstream tumor-suppressive targets PTEN and PDCD4. This may further lead to miR-21 being used as a biomarker and cancer treatment target to improve prognosis in OSCC patients (Liu et al., 2017).

## LncRNA

LncRNAs are also packaged in exosomes (Fang et al., 2020). Exosome lncRNA mainly acts as messengers in intercellular communication and participates in the regulation of the cell microenvironment. Dysregulation of exosomal lncRNA will affect angiogenesis, metastasis, and drug resistance, thus promoting the occurrence and development of tumors (Xu et al., 2018). Oral submucosal fibrosis (OSF) is a precancerous lesion of OSCC (Mithani et al., 2007). Zhou and others investigated the role of a disintegrin and metalloproteinase with thrombospondin motifs (ADAMTS) family protein members in OSF carcinogenesis. They found that lncRNA ADAMTS9-AS2 was significantly up-regulated in normal oral mucosa tissues, but down-regulated in OSCC and OSF tissues, and low expression was associated with poor prognosis. Further study of the mechanism revealed that lncRNA ADAMTS9-AS2 inhibited PI3K-Akt signaling pathway, regulated EMT, and suppressed proliferation and metastasis of OSCC cells. It highlights the key role of exosomal lncRNA ADAMTS9-AS2 in OSF carcinogenesis and is expected to be a biomarker for the early diagnosis of OSCC (Zhou et al., 2021). Zinc finger antisense 1 (ZFAS1), as a lncRNA, was demonstrated by Wang et al. to be up-regulated in serum exosomes of OSCC. As a result of upregulation, OSCC cells increased proliferation and inhibited the sensitivity of OSCC cells to cisplatin. Specifically, overexpressed ZFAS1 inhibited transcription by down-regulating the expression of miR-421, thereby increasing myeloid ecotropic viral integration site 1 homolog 2 (MEIS2) expression, ultimately leading to the proliferation and promotion of chemical resistance to OSCC. The ZFAS1/miR-421/MEIS2 pathway regulates OSCC proliferation and chemoresistance to cisplatin and may serve as a promising therapeutic target in OSCC (Wang et al., 2020). Tumor cells and CAF can communicate directly and effectively through the generation of lncRNA-rich exosomes (Dai et al., 2020). Ding et al. found that in OSCC cells, exosomes containing lncRNA-CAF (lnc-CAF) could be secreted into fibroblasts, thereby secreting more lnc-CAF to activate CAF, forming a positive feedback loop and promoting the proliferation of OSCC cells (Ding et al., 2018).

## CircRNA

CircRNA is rich in exosomes. The covalent closed-loop structure of circRNA endows circRNA with high stability and unique molecular conformation. *In vitro* synthesis of circRNA also has potential immunogenicity (Meng et al., 2017). CircRNA can be used as miRNA sponge, competitive binding protein or protein scaffold to serve as a tool for cell function detection and manipulation of intracellular processes, to enhance the efficiency of response and protein translation. These characteristics make it a good diagnostic biomarker or therapeutic target for HNSCC (Li et al., 2018; Bai et al., 2019; Slack and Chinnaiyan, 2019). Luo et al. demonstrated that overexpressed circ_0000199 in circulating exosomes was significantly associated with areca chewing, tumor size, lymph node metastasis, and TNM staging in OSCC patients. Tumor recurrence and mortality were also higher in OSCC patients with low exosome circ_0000199. It was further found by gain and loss function experiments that overexpression of circ_0000199 could promote cell proliferation and inhibit apoptosis, while knockdown of circ_0000199 showed the opposite effect. ECM-receptor interaction, transforming growth factor-β (TGF-β) signaling pathway, and MAPK signaling pathway were mainly downstream signaling pathways, which regulated the proliferation and apoptosis of OSCC cells. These results suggest that highly expressed circulating exosome circ_0000199 can be an independent predictor of survival and disease recurrence in OSCC patients, but the specific regulatory mechanisms remain to be further studied (Luo et al., 2020). Tian et al. found that serum exosome circRASSF2 was highly expressed in LSCC cells. Moreover, it was verified that circRASSF2 promotes the development of LSCC through its sponge effect on miR-302b-3p. Western blotting confirmed that circRASSF2 inhibition reduced insulin-like growth factor 1 receptor (IGF-1R) expression. Thus, it is verified that serum exosome circRASSF2 caused the malignant progression of LSCC through circRASSF2/miR-302b-3p/IGF-1R axis (Tian et al., 2019).

Although growing evidence has shown that bioactive molecules in the exosomes of HNSCC can be used as potential non-invasive biomarkers for detection and monitoring of HNSCC, the results are still not validated for clinical practice in HNSCC subtypes (Bhat et al., 2021). In order to find more possible prognostic and early screening markers, sensitivity and specificity are crucial, and it is necessary to address the cross-reactivity of multiple exosome markers and interference of nanoparticles (Cheng et al., 2019). Most studies have focused on the mechanism of a single signaling pathway mediated by exosomes containing certain proteins and RNAs. Other exosomal cargoes (DNA, lipids, and metabolites) need to be searched and verified to further study the complex regulatory network on TME, determine the overall function of a single bioactive component and the synergistic function of multiple bioactive components and verify driving factors and causal relationships (Yáñez-Mó et al., 2015; Fang et al., 2020). In addition, the scarcity of circulating biomarkers and their relative instability in circulation is also an urgent problem to be solved (Hofmann et al., 2020a). In conclusion, further studies are needed to deepen the understanding of the complex functions of exosomes, clarify the exact mechanisms of biomarkers, and develop technical standards to improve therapeutic outcomes in patients with HNSCC (Yang et al., 2020a). (Table 2 summarizes the comparison of features and role in tumor of miRNA, lncRNA and circRNA. Table 3 summarizes the studies on biomarkers of exosomes in HNSCC patients.)

**TABLE 2:** Comparison of features and role in tumor of miRNA, lncRNA and circRNA.

	Features	Role in tumor	Refs
miRNA	a. NcRNAs of approximately 22 nt in length	a. Translation inhibition	Krol et al. (2010); Ryan et al. (2010); Pallante et al. (2014); Slack and Chinnaiyan (2019)
	b. Not easy to degrade, with high stability	b. Degradation of mRNA	—
	c. Highly conservative	c. Transcriptional regulation	—
	d. Timing expression specificity	—	—
	e. Tissue expression specificity	—	—
	f. The regulation is not very strong	—	—
lncRNA	a. NcRNAs greater than 200 nt in length	a. Regulation of transcription levels	Hart and Goff (2016); Slack and Chinnaiyan (2019); Alessio et al. (2020)
	b. No or weak protein coding ability	b. Regulation of the level of epigenetic modification	—
	c. Low conservatism	c. Regulation of post-transcriptional levels	—
	d. Timing expression specificity	—	—
	e. Tissue expression specificity	—	—
circRNA	a. High stability	a. Regulation of transcription, splicing, and chromatin interactions	Li et al. (2015); Qu et al. (2015); Slack and Chinnaiyan (2019)
	b. Highly conservative	b. A miRNA sponge	—
	c. Timing expression specificity	c. Acting as a protein scaffold	—
	d. Tissue expression specificity	d. Competitive binding proteins	—

**TABLE 3:** Biomarkers of HNSCC exosomes.

Exosome source	Bioactive substance	Outcome	Application	Refs
OSCC	HSP90↑	Metastasis ↑	P	Ono et al. (2018)
HNSCC	LOXL2↑	Early HNSCC ↑	D	Sanada et al. (2020)
HNSCC	ANXA1 ↓	Proliferation and invasion ↑	P	Raulf et al. (2018)
OSCC	Alix ↑	Metastasis and tumor stage ↑	D&P	Nakamichi et al. (2021)
OSCC	miR-365 ↑	Liquid biopsy	D	Coon et al. (2020)
HPV (+) HNSCC	miR-9 ↑	Radiosensitivity ↑	P	Tong et al. (2020)
LSCC	miR-941 ↑	Proliferation and invasion ↑	D	Zhao et al. (2020b)
OSCC CAFs	miR-382-5p ↑	Proliferation and invasion ↑	P	Sun et al. (2019)
OSCC CAFs	miR-14 ↑	Proliferation and invasion ↑	P	Kozłowska et al. (2021)
OSCC	miR-21 ↑	Proliferation and invasion ↑	D&P	Li et al. (2016)
OSCC	miR-21 ↑	Cisplatin resistance ↑	P&C	Liu et al. (2017)
OSCC	miR-24-3p ↑	Proliferation ↑	D	He et al. (2020)
HNSCC	miR-196a ↑	Tumor size, metastasis, tumor stage, and chemical resistance ↑	P&C	Qin et al. (2019)
OSCC	lncRNA ADAMTS9-AS2 ↓	Proliferation, migration and invasion ↑	D&P	Zhou et al. (2021)
OSCC	ZFAS1↑	Proliferation/cisplatin resistance ↑	P&C	Wang et al. (2020)
OSCC	circ_0000199 ↑	Tumor size, metastasis, and tumor stage ↑	P	Luo et al. (2020)
LSCC	circRASSF2 ↑	Proliferation, migration and invasion ↑	P	Tian et al. (2019)

(P=Prognosis D = Diagnosis C=Chemical resistance).

## Exosomes as Drug Delivery Vectors

Compared with free drug delivery in mouse models, exosome-based drug delivery has better anti-tumor effects (Wang et al., 2017). Exosomes are being actively explored as suitable drug delivery vectors or therapeutic agents because of their molecular structure advantages (Batrakova and Kim, 2015). First, clinically widely used blood transfusions involve injections of more than one trillion other exosomes, but do not exhibit immune-related toxicity in recipients. Exosome injection of allogeneic exosomes may not cause major complications, reflecting the low immunogenicity of exosomes (Kalluri and LeBleu, 2020). Exosomes can then lead to surface lipid composition and protein content with inherent targeting properties. This can be used to design ligand enrichment on exosomes, induce or inhibit signaling events in recipient cells, or target exosomes to specific cell types (Murphy et al., 2019; Zhao et al., 2020a). Finally, exosomes but also have good biological compatibility, easy to produce and store, non-toxic, long shelf life, and high load capacity. These advantages make exosomes unresponsive to patients with conventional or drug-resistant HNSCC for targeted delivery of metastatic recurrence and chemotherapy drugs, reducing cytotoxicity. It could be used as a potential drug delivery tool to treat HNSCC (Srivastava et al., 2016; Wang et al., 2017; Gupta et al., 2021). Currently, clinical trials of chemotherapy-loaded exosomes as anticancer drug delivery systems are increasing. Other cancers already have carriers of chemotherapeutic drugs, such as doxorubicin (DOX), curcumin, and paclitaxel (PTX), which have shown promising performance in improving therapeutic efficacy and reducing side effects (Ketabat et al., 2019). As an exogenous cancer treatment, electroporation, co-incubation, or ultrasound can be used to deliver chemotherapeutic drugs into exosomes. Endogenous methods, which rely on cellular mechanisms, can spontaneously embed drugs through a continuous process from cell isolation to chemical drug incubation (Walker et al., 2019). Cui et al. reported that exosomes of normal tongue epithelial cells overexpressing miR-200c could deliver miR-200c to PTX resistant tongue squamous cell carcinoma (TSCC) cells *in vitro*, increasing sensitivity to PTX treatment. *In vivo*, intratumor injection of overexpressing exosome miR-200c significantly inhibited the growth of TSCC in response to PTX treatment. MiR-200c reduced the PTX resistance of PTX-resistant TSCC cells mainly by targeting TUBB3 and PPP2R1B. Therefore, exosome-mediated miR-200c delivery may be an effective and promising strategy for regulating TSCC chemical resistance (Cui et al., 2020).

In summary, ligands and adhesive proteins in exosomes bind to cell membranes, making exosomes excellent carriers for targeted drug delivery. As it stands, the application of exosomes derived from human tissues as drug delivery tools for performing specific therapies in the context of personalized medicine takes full advantage of exosomes as natural carriers, but some key issues still need to be addressed. Although they have encouraging preclinical evidence for cancer therapy, the ability of high-purity exosomes to deliver high-dose therapeutic drugs is limited and methods for isolating high-purity exosomes need to be improved. It is necessary to address the differences in drug uptake by different exosomes in different target tissues and to determine the optimal dose, delivery method, and kinetic properties of the drug. Furthermore, studies have shown that exosome administration in patients may lead to adverse immune reactions (Ha et al., 2016; Luan et al., 2017). In response to the complexity of exosomes, it has been proposed that the exosomes of therapeutic drugs can be internalized by incorporating cell-penetrating peptides on the surface of microcytosis-interacting cells at the target site (Jan et al., 2021). It is also important to explore the therapeutic response (Yang et al., 2020a). These aspects limit the use of exosomes as an effective drug delivery system and further research is needed to advance progress.

## Exosomes in Tumor Immunotherapy

The immune activity of exosomes plays an immunomodulatory role in antigen presentation, immunomodulatory monitoring, immunomodulatory activation, and inhibition (Xiao et al., 2019). As a highly immunosuppressive malignancy, exosomes in HNSCC contain mainly immunosuppressive molecules, which help cancer cells to evade immune responses and advance the progression of immunosuppression in HNSCC (Filipazzi et al., 2012; Monypenny et al., 2018). Exosomes derived from different cell sources play different roles in tumor immunity. Exosomes derived from tumor cells and immune cells are abundant in plasma of HNSCC patients (Ludwig et al., 2017; Theodoraki et al., 2018a). TEX mainly reflects the tumor status of HNSCC, while exosomes produced by immune cells mainly reflect the immune dysfunction of HNSCC (Whiteside, 2018; Whiteside, 2019; Hofmann et al., 2020b). Although the specific mechanisms by which tumor exosomes regulate host immunity are complex and largely unknown, some recent research results on HNSCC are trying to explain the great heterogeneity of immune regulation mechanisms (Xie et al., 2019).

## Immune Cells

In HNSCC, exosomes secreted by various immune cells including T cells, macrophages, and monocytes extensively regulate T cell function and antigen presentation mainly by exerting an immunosuppressive effect, thus leading to efficient immunosuppression (Lugini et al., 2012; Liu et al., 2016; Saunderson and McLellan, 2017; Xie et al., 2019). Although the current research on immune cell exosomes is far behind compared with tumor cell exosomes, it is of great significance to understand the function of immune cells, especially in exploring the composition, characteristics, and functional proteins of different immune cell exosomes (Xie et al., 2019). CD3 (+) exosomes derived from T cells carry immunomodulatory molecules that inform parental T cell function and correlate closely not only with clinicopathological parameters but also significantly with immunotherapeutic responses (Theodoraki et al., 2018a; Theodoraki et al., 2019). Studies assessed the predictive value of plasma derived HNSCC CD3 (+) exosomes as T lymphocyte substitutes. Enrichment and isolation of exosomes secreted by CD3 (+) T cells from CD3 (-) TEX by immunocapture revealed higher levels of CD3 (+) exosomes in patients who responded to treatment. This finding was mainly related to stronger T-cell activation and lower level of immunosuppression in patients (Theodoraki et al., 2019). Hofmann and others found CD16 on the surface of plasma exosomes from tumor patients and significantly elevated levels of CD16 in the exosomes of HNSCC patients. Because the plasma exosome population represents a mixture of exosomes from different cell types, while TEX showed some levels of surface CD16, total exosomes, representing all cell populations in TME, showed higher levels. In addition, total exosome CD16 levels were significantly higher in patients with advanced-stage T3/4 tumors and UICC III/IV HNSCC than in patients with early-stage T1/2 tumors and UICC I/II HNSCC. This was not only significantly correlated with tumor stage and invasion but may even be an indicator of HNSCC immunosuppression grade (Hofmann et al., 2020b). The results of Hofmann and other laboratory studies emphasized the expression of CD16 in monocyte subpopulations, although it was not possible to distinguish which immune cell population CD16 positive exosomes come from, based on current data (Yeap et al., 2016). Bellmunt and his team determined that macrophage exosome-mediated signaling enhances LSCC cell migration using a transwell system and scratch assay. This study also found that exosomes could also enhance the immunosuppressive state by inducing IL-10 expression in LSCC macrophages (Bellmunt et al., 2019).

## TEX

Immune cells infiltrate tumor tissue and interact with tumor and stromal cells in TME. Tumor cells can secrete TEX to deliver immune-stimulating or immunosuppressive signal molecules, and target to regulate the development, maturity, and anti-tumor ability of the immune system (Robbins and Morelli, 2014; Whiteside, 2016b; Xie et al., 2019). However, in most cases, TEX acts as an immunosuppressant in HNSCC (Xie et al., 2019). TEXs can carry a variety of tumor antigens, and TEXs play an important role in immunosuppressive mediators and are considered to be one of the key immunosuppressive mechanisms in TME (Hofmann et al., 2020a; Sharma et al., 2020). Beccard et al. found that although CD45 (+) exosomes had immunosuppression potential in HNSCC, the highest immunosuppression was caused by TEX. CD45 (-) exosomes were highly enriched in TEX. CD45 (-) exosomes were significantly higher in HNSCC stage III/IV patients than in HNSCC stage I/II patients, inducing more apoptosis. This might be because high levels of CD45 (-) HNSCC exosomes had higher stage-dependent variability in immunosuppressive molecular cargo compared to CD45 (+) exosomes with higher static molecular cargo. It might also be reflected in the high inhibition of CD69 on activated CD8 (+) T cells. CD45 (-) presentation showed that TEX significantly induces immunosuppression in HNSCC (Beccard et al., 2020). It had also been reported that TEX secreted CD44v3 (+) exosomes rich in Programmed death ligand 1 (PD-L1), human factor related apoptosis ligand (FasL), TGF-β, and EGFR proteins, promoting the growth and immunosuppression of HNSCC. At the same time, the relative fluorescence intensity values of these proteins were significantly increased in stage III/IV patients compared to HNSCC stage I/II patients (Hofmann et al., 2020b).

The incidence of HNSCC caused by human papillomavirus (HPV) infection is increasing. Patients with HPV(+) tumors respond well to initial therapy, so HPV has become an area of HNSCC immune research (Taberna et al., 2017). Ludwig et al. found that only exosomes released by HPV(+) HNSCC cells contained viral proteins E6/E7, P16, and survivin. These exosomes also carried costimulant OX40, OX40L, and HSP70 molecules, which produced strong stimulants in human T lymphocyte assays, leading to strong immune responses to viral antigens. These results suggest that exosomes released by HPV(+) HNSCC cells may play a role in mediated immune activation in anti-tumor immune response, providing a method to improve the sensitivity of conventional tumor therapy (Ludwig et al., 2018). In addition, HPV(+) HNSCC cells contained immune-effector cell-associated antigens CD47 and CD276. CD47 protected tumors by sending inhibitory signals to macrophages and other cells *via* SIRPα to inhibit phagocytosis. CD276, as a coinhibitory molecule, also played a similar role. In addition to the discovery that HPV(+) exosomes were rich in CD47 and CD276, it was also found by proteomics that HPV(-) exosomes contained negative modulators of immune response MUC-1 and HLA-DRA, which played a crucial role in anti-tumor defense. The difference in immune response between HPV(+) type and HPV(-) HNSCC is due to the protein content. This provides a possible explanation for the higher resistance and poor prognosis of HPV(-) HNSCC (Ludwig et al., 2019).

As a membrane-binding ligand on many cancer cells, PD-L1 can bind the programmed death 1 (PD-1) receptor on T cells, inhibiting the antigen-derived activation of T cells and triggering immune checkpoint responses (Yokosuka et al., 2012; Hui et al., 2017). Previous studies have found that PD-L1 has been found in TEXs in plasma samples of patients with various cancers (Theodoraki et al., 2018b). Other study found that plasma PD-L1 (+) exosomes in HNSCC patients inhibited T cell activation by driving CD69 expression on the PD-1/PD-L1 down-regulation signal on T cells. It is proved that circulating PD-L1 (+) exosomes can induce immune dysfunction in HNSCC patients by inducing T cell dysfunction. In addition, HNSCC patients with disease activity or advanced exosome PD-L1 levels have a status that the higher the exosome PD-L1 level, the stronger the inhibitory effect on T cell activity. The level of exosome PD-L1 is significantly positively correlated with the progression of HNSCC, lymph node involvement, and high tumor stage (Theodoraki et al., 2018b).

Although exosome-based strategies have been shown to enhance anti-cancer immunotherapy, they are still in the early stages of clinical trials and are still some ways from reaching the clinical stage (Li et al., 2020). The specific mechanisms of exosomes in immune regulation are complex and largely unknown, but it is essential to distinguish between relapse and increased levels of exosomes due to inflammation after immunotherapy, as exosomes are involved in both pathways and have an important identity. In addition, further proof is needed of whether targeted therapies with exosomes have a preventive effect on tumor metastasis (Yang et al., 2020b). These will further investigate the effect of exosomes on the immune system and may reveal yet undiscovered mechanisms underlying the suppression of antitumor immunity, leading to the discovery of novel immune targets for drug therapy (Clayton et al., 2020). Guidelines or international guidelines for the new treatment also need to be developed (Zhang and Yu, 2019). (Table 4 summarizes the studies on immunosuppression of HNSCC exosomes.).

**TABLE 4:** Immunosuppression of HNSCC exosomes.

Exosome source	Bioactive substance	Function	Refs
HNSCC	CD3 (+)	Levels of CD3 (+) exosomes were higher in patients who responded to treatment. It is mainly related to the strong T cell activation ability and low immunosuppression level of patients	Theodoraki et al. (2019)
HNSCC	CD16	The level of total exosome CD16 in HNSCC patients was significantly correlated with tumor stage and invasion, indicating that the later the stage was, the greater the immunosuppressive effect was	Hofmann et al. (2020b)
HNSCC	CD45 (-)	CD45 (-) exosomes, highly enriched in TEX, not only correlated with stage but also induced more apoptosis, thus inducing immunosuppression of HNSCC.	Beccard et al. (2020)
HNSCC	CD44v3 (+)	TEX secreted CD44v3 (+) exosomes rich in PD-L1, FasL, TGF-β and EGFR proteins, promoting HNSCC progression and immunosuppression	Hofmann et al. (2020b)
HNSCC	MUC-1 and HLA-DRA	Negative regulators of immune responses in HPV(-) exosomes included MUC-1 and HLA-DRA. MUC-1 (+) exosomes protect HNSCC cells from activated NK cell-mediated lysis. HLA-DRA was the ligand of T cell receptor, and its signal transduction promoted the production of Treg	Ludwig et al. (2019)
HNSCC	CD69	Plasma PD-L1 (+) exosomes in HNSCC patients inhibited T cell activation by driving CD69 expression of down-regulation PD-1/PD-L1 signaling for T cells	Theodoraki et al. (2018b)

## Conclusion and Prospects

New evidence is being presented for the importance of exosomes as multifunctional carriers of intercellular communication in HNSCC. Exosomes secreted by HNSCC cells and their surrounding stromal cells mix and communicate with recipient cells and participate in metabolic reprogramming and microenvironmental remodeling, leading to metabolic changes (Yang et al., 2020b). Exosomes can be used as an ideal diagnostic and prognostic biomarker for HNSCC due to their unique secretion pattern. Exosomes can be directly involved in the anti-tumor process of drugs, and can also be transformed into transport vectors of anti-tumor substances *in vitro*, and can also be used as immune inducers to induce specific anti-tumor immune responses (Jang et al., 2013; Armstrong and Stevens, 2018; Chulpanova et al., 2018). However, exosomes that intend to be used as diagnostic or therapeutic agents in the future still are required to elucidate the molecular mechanisms of exosome production, endocytosis, and biological roles in tumor progression, based on larger patient cohorts (Dai et al., 2020; Ebnoether and Muller, 2020). Standardized methods for the selection of exosome sources and isolation techniques are currently being explored to achieve more and pure production of exosomes with unique characteristics and functions (Hofmann et al., 2020a; Yang et al., 2020a). The main frustration of exosome research is the lack of in-depth research on the underlying biology. Therefore, the application as a drug delivery system and targeted drug therapy is delicate. Because the exact changes and interactions of exosomes as therapeutic vesicles are not known (Ebnoether and Muller, 2020). There are still many immature areas in the field of exosomes, but it is undeniable that it greatly promotes the occurrence, proliferation, metastasis, immunosuppression and chemotherapy resistance of HNSCC, opening a window of hope for the fight against HNSCC. The potential is enormous.

## Glossary

ADAMTS: a disintegrin and metalloproteinase with thrombospondin motifs
ANXA1: Annexin A1
CAFs: cancer-associated fibroblasts
circRNA: circular RNA
DOX: doxorubicin
EBV: Epstein-Barr Virus
EGFR: epidermal growth factor receptor
EMT: epithelial-mesenchymal transition
ESEs: early sorting endosomes
ESCRT: endosomal-sorting complex required for transport
EVs: extracellular vesicles
FasL: human factor related apoptosis ligand
HNSCC: head and neck squamous cell carcinoma
HPV: human papillomavirus
IDO: indoleamine 2,3 dioxygenase
IGF-1R: insulin-like growth factor 1 receptor
ILVs: intraluminal vesicles
Lnc-CAF: lncRNA-CAF
lncRNA: long non-coding RNA
LOXL2: lysyl oxidase-like 2
LSEs: late sorting endosomes
LSCC: laryngeal squamous cell carcinoma
MEIS2: myeloid ecotropic viral integration site 1 homolog 2
miRNA: microRNA
MVB: multivesicular body
ncRNAs: non-coding RNAs
OSCC: oral squamous cell carcinoma
OSF: oral submucosal fibrosis
PD-1: programmed death receptor 1
PD-L1: Programmed death ligand 1
PTX: paclitaxel
TEXs: tumor-derived exosomes
TGF-β: transforming growth factor-β
TME: tumor microenvironment
TNM: tumor-node-metastasis
TSCC: tongue squamous cell carcinoma
ZFAS1: zinc finger antisense 1
