# Corrigendum: Exosomes: Potential biomarkers and functions in head and neck squamous cell carcinoma

**DOI:** 10.3389/fmolb.2022.1056179

**Published:** 2022-11-03

**Authors:** Ting Li, Juan Li, Haitao Wang, Jiayu Zhao, Mingze Yan, Hongjiang He, Shan Yu

**Affiliations:** ^1^ Department of Head and Neck Surgery, Harbin Medical University Cancer Hospital, Harbin, China; ^2^ Department of Pathology, Second Affiliated Hospital of Harbin Medical University, Harbin, China; ^3^ Thoracic Surgery Branch, National Cancer Institute, National Institutes of Health, Bethesda, MD, United States

**Keywords:** head and neck squamous cell carcinoma, exosome, biomarkers, therapeutic target, drug carrier

In the published article author “Hongjiang He” was erroneously not attributed as a corresponding author.

The authors apologize for this error and state that this does not change the scientific conclusions of the article in any way. The original article has been updated.

